# The Mitochondrial Basis of Aging and Age-Related Disorders

**DOI:** 10.3390/genes8120398

**Published:** 2017-12-19

**Authors:** Sarika Srivastava

**Affiliations:** Virginia Tech Carilion Research Institute, 2 Riverside Circle, Roanoke, VA 24016, USA, Sarika_Srivastava@vtc.vt.edu; Tel.: +1-540-526-2047

**Keywords:** mitochondria, mitochondrial dysfunction, aging, reactive oxygen species, mitochondrial dynamics, mitophagy, mitochondrial biogenesis, age-related disorders

## Abstract

Aging is a natural phenomenon characterized by progressive decline in tissue and organ function leading to increased risk of disease and mortality. Among diverse factors that contribute to human aging, the mitochondrial dysfunction has emerged as one of the key hallmarks of aging process and is linked to the development of numerous age-related pathologies including metabolic syndrome, neurodegenerative disorders, cardiovascular diseases and cancer. Mitochondria are central in the regulation of energy and metabolic homeostasis, and harbor a complex quality control system that limits mitochondrial damage to ensure mitochondrial integrity and function. The intricate regulatory network that balances the generation of new and removal of damaged mitochondria forms the basis of aging and longevity. Here, I will review our current understanding on how mitochondrial functional decline contributes to aging, including the role of somatic mitochondrial DNA (mtDNA) mutations, reactive oxygen species (ROS), mitochondrial dynamics and quality control pathways. I will further discuss the emerging evidence on how dysregulated mitochondrial dynamics, mitochondrial biogenesis and turnover mechanisms contribute to the pathogenesis of age-related disorders. Strategies aimed to enhance mitochondrial function by targeting mitochondrial dynamics, quality control, and mitohormesis pathways might promote healthy aging, protect against age-related diseases, and mediate longevity.

## 1. Introduction

Aging is broadly defined as a time-dependent gradual and progressive decline in living organisms cellular and organ functions leading to increased vulnerability to chronic diseases and death [1,2]. Nine candidate hallmarks of mammalian aging have recently been identified and classified under three categories i.e., the primary hallmarks (genomic instability, telomere attrition, epigenetic alterations, and loss of proteostasis), the antagonistic hallmarks (mitochondrial dysfunction, deregulated nutrient sensing and cellular senescence), and the integrative hallmarks (stem cell exhaustion and altered intercellular communication) [2]. The primary hallmarks are underlying cause of molecular damage during aging, the antagonistic hallmarks exert beneficial or protective effects at low levels but are deleterious to the organism at high levels, and the integrative hallmarks arise when the cellular homeostatic mechanisms fail to compensate for the accumulating damage [2,3]. Importantly, the aging hallmarks interconnect and impinge upon cellular metabolism, thus targeting metabolism may be a promising strategy towards extending human healthspan and lifespan [3].

Mitochondria are central to regulating cellular metabolism and homeostasis due to their key roles in bioenergetics, generation of reactive oxygen species (ROS), anabolism and catabolism, iron–sulfur cluster and heme biosynthesis, calcium and iron homeostasis, apoptosis and signal transduction [4,5,6,7,8]. These organelles are vital for life, dynamic and reprogram metabolism in response to cellular stress [5,9]. Mitochondrial dysfunction is linked to various aspects of aging including impaired oxidative phosphorylation (OXPHOS) activity, increased oxidative damage, decline in mitochondrial quality control, reduced activity of metabolic enzymes, as well as changes in mitochondrial morphology, dynamics and biogenesis [10,11]. Mitochondrial dysfunction is also implicated in numerous age-related pathologies including neurodegenerative and cardiovascular disorders, diabetes, obesity and cancer [7,8,12,13,14,15,16,17,18]. The maintenance of mitochondrial and cellular homeostasis requires a tight regulation and coordination between generation of new and removal of damaged mitochondria. The damaged or dysfunctional mitochondria are selectively degraded by a mitochondria-specific autophagy clearance process known as mitophagy, whereas new mitochondria are synthesized by mitochondrial biogenesis. An intricate regulatory network balances the mitophagy and mitochondrial biogenesis processes, thus proper coordination of these two opposing processes is critical for aging and longevity [19,20,21]. Dysfunctional or stressed mitochondria generate signals that trigger activation of mitochondrial-to-nuclear, mitochondrial-to-cytosolic, and non-cell-autonomous responses that act to protect against cell death, and restore cellular and metabolic homeostasis [9,22,23]. Here, I will provide a comprehensive overview on the role of mitochondrial DNA (mtDNA) mutations, ROS, oxidative stress, mitochondrial fission, fusion, biogenesis and turnover pathways in aging. Further, I will discuss the emerging evidence that an imbalance between mitochondrial fission and fusion as well as mitochondrial degradation and biogenesis pathways contribute to the pathogenesis of various age-related disorders. Therapeutic strategies that improve mitochondrial dysfunction by targeting mitochondrial dynamics, quality control and stress response-mediated mitohormesis pathways may benefit humans in terms of healthy aging, protection against age-related diseases, and longevity.

## 2. Mitochondria, Aging and Age-Associated Pathologies

Mitochondria are vital for life as these organelles serve as the powerhouse or energy currency of the eukaryotic cells. Mitochondria generate energy or adenosine triphosphate (ATP) by breakdown of fuel (i.e., glucose and fatty-acids) through a series of redox reactions performed by a set of five electron transport chain (ETC) enzyme complexes of the mammalian OXPHOS system [24]. The role of mitochondria in aging was first proposed more than 40 years ago by Denham Harman, who postulated the free radical theory of aging, suggesting that accumulation of cellular damage with increasing age results from reactive oxygen species (ROS) and mitochondria are one of the most important sources and targets of ROS that could function as an ‘aging clock’ [25,26]. Since then, a growing body of evidence has shown that mitochondrial dysfunction contributes to aging in multiple model organisms and that several factors cause increased mitochondrial dysfunction with chronological age including accumulation of somatic mtDNA mutations, enhanced oxidative damage, decreased abundance and quality of mitochondria, as well as dysregulation of mitochondrial dynamics as discussed below.

### 2.1. Somatic Mitochondrial DNA Mutations

**Figure 1:**
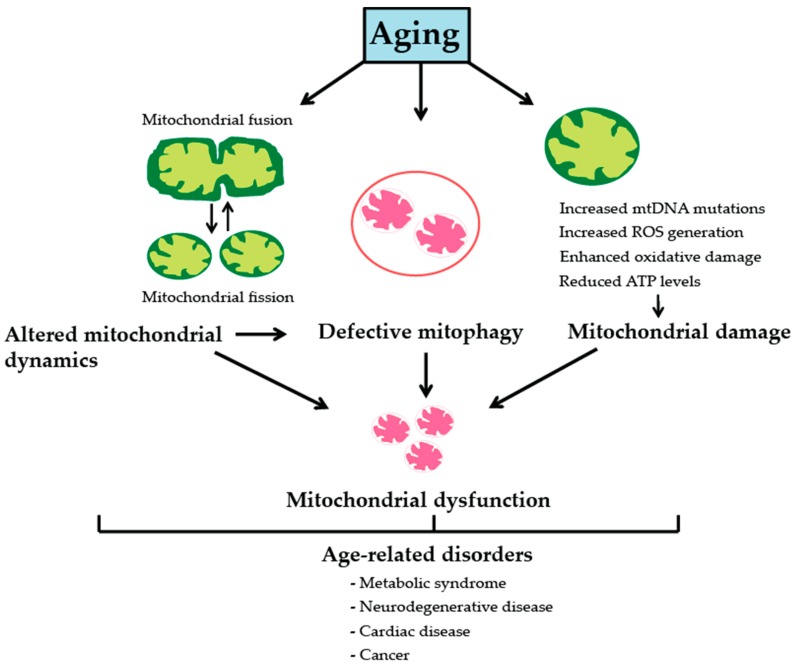
Mitochondrial dysfunction during aging and age-related disorders. Aging is associated with progressive mitochondrial dysfunction that occurs due to accumulation of mitochondrial DNA (mtDNA) mutations and increased reactive oxygen species (ROS) production that causes oxidative damage to cellular macromolecules, thereby leading to reduced respiratory chain activity and adenosine triphosphate (ATP) generation. Mitochondrial fission and fusion play a vital role in the regulation of mitochondrial function, metabolism and quality control. Altered mitochondrial dynamics with chronological age can inhibit mitophagy leading to accumulation of damaged or dysfunctional mitochondria in cells. Moreover, decline in mitophagy with increasing age prevents clearance of dysfunctional mitochondria leading to further mitochondrial damage accrual and deterioration of cellular function. Genetic mutations or functional declines in mitochondrial dynamics and quality control are thus linked to pathogenesis of numerous age-related disorders including metabolic syndrome, neurodegenerative and cardiovascular diseases as well as cancer.

Mitochondria are unique as they harbor their own genome (mtDNA), which in humans is ≈16,569 base pairs (bp) double-stranded circular molecule containing 37 genes that encode 13 messenger RNAs (mRNAs), 22 transfer RNAs (tRNAs) and 2 ribosomal RNAs (rRNAs) [24]. Point mutations and deletions are the two most frequent types of mutations that arise in mtDNA genome with age mainly due to spontaneous errors during mtDNA replication or damage repair. A wealth of supportive evidence demonstrates that mitochondrial dysfunction occurs with age due to accumulation of mtDNA mutations (Figure 1); however, the causative role of mtDNA mutations in aging remains controversial. Various mtDNA point mutations have been shown to significantly increase with age in the human brain, heart, skeletal muscles and liver tissues [27,28,29,30,31,32]. Increased frequency of mtDNA deletions/insertions have also been reported with increasing age in both animal models and humans [33,34]. MtDNA deletion mutations were shown to accumulate intracellularly to high levels in aged human skeletal muscle fibers and associate with segmental ETC abnormalities, fiber atrophy, splitting and enhanced oxidative damage leading to sarcopenia [35,36]. Accumulation of mtDNA 4977 bp deletion (common deletion) was also shown to occur with increasing age (from 39 to 82 years) in different brain regions of normal human adults [37]. The age-associated mosaic respiratory chain deficiency in the forebrain of a chimeric mouse that harbored a mixture of normal and respiratory chain-deficient neurons revealed that low proportion (>20%) of ETC deficiency was sufficient to induce neuronal mitochondrial dysfunction and neurological disease [38]. Moreover, an increase in the number of neurons with cytochrome c oxidase (COX) deficiency was reported in the substantia nigra and hippocampus of normal human aging brains indicating that complex IV defects significantly increase with age causing mitochondrial dysfunction [32,39,40]. Loss of COX activity was also reported to occur physiologically with age in various other tissues including heart, skeletal muscles, diaphragm and colonic crypt stem cells further supporting a role of mitochondrial dysfunction in aging [29,30,41]. The strongest evidence to date that favors a causative role of mtDNA mutations in aging comes from the study of mtDNA mutator mice that express a proofreading exonuclease activity deficient version of mtDNA polymerase γ (Polg^mut/mut^) and exhibit significant accumulation of mtDNA mutations as well as premature or accelerated aging phenotype [42,43]. 

Although accumulation of somatic mtDNA mutations has been implicated as a causal factor in aging by numerous studies, there are multiple conflicting reports raising the controversy whether mtDNA mutations accumulate to sufficiently high levels with age to establish their causative role in the aging process [27,44,45]. MtDNA exists in multiple copies (i.e., hundreds to thousands) per cell and based on the phenotypic expression threshold the mtDNA mutation load (or heteroplasmy) exceeding a critical threshold (i.e., >60–80% heteroplasmy based on the type of mutation) would lead to mitochondrial dysfunction and disease expression [46]. Notably, the abundance of mtDNA mutations rarely exceeds ≈1% with age in normal human adults which is well below the phenotypic expression threshold [32,47]. The idea that mtDNA deletions cause aging and age-related diseases was previously contradicted by the study of mito-mice harboring ≈4.7 Kbp mtDNA deletion uniformly distributed among various tissues and showing that an accumulation of up to ≈60% mtDNA deletion in different tissues displayed no signs of mitochondrial dysfunction and disease manifestation, whereas tissues with >85% mtDNA deletion exhibited mitochondrial dysfunction and disease phenotypes [48,49]. Another study that strongly argued against the possible role of mtDNA mutations in the aging process employed a highly sensitive random mutation capture assay to show that mtDNA mutation frequency in the brain and heart tissues of wild-type aged mice was ≈10 times lower than previously reported, which is in striking contrast to the mtDNA mutation load that is ≈500-fold higher in mtDNA mutator heterozygous mice (Polg^mut/+^) that exhibit a normal life span and no signs of premature aging [50]. However, the caveats of this study are that their assay did not detect any large-scale mtDNA deletions which have been shown to accumulate in the aged human tissues and that any species-specific differences could not be ruled out which may causally relate mtDNA mutations to aging in humans [40,44]. Further studies are required to clarify any species-specific differences in somatic mtDNA mutation accumulation during aging and to determine whether relatively lower mtDNA mutation frequency reported in aged mice may be functionally relevant or irrelevant for human aging.

### 2.2. Reactive Oxgygen Species and Oxidative Stress

Reactive oxygen species are an important byproduct of oxidative metabolism that comprises of superoxide anions (O_2_^•−^), hydrogen peroxide (H_2_O_2_) and hydroxyl (^•^OH) radicals. Single electron reduction of molecular O_2_ generates O_2_^•−^ anions which are rapidly converted by superoxide dismutases (SODs) to produce H_2_O_2_, whereas in the presence of reduced transition metals the H_2_O_2_ is converted to highly reactive ^•^OH radicals by Fenton reaction [51,52]. ROS spontaneously oxidizes nucleic acids, lipids and proteins causing macromolecular damage [53,54,55]. The free radical theory of aging postulates that progressive mitochondrial dysfunction with chronological age increases ROS production, which in turn causes oxidative damage to cellular macromolecules, thereby initiating a ‘vicious cycle’ of ROS generation and molecular damage accumulation [25,26]. An imbalance between excessive ROS production and limited cellular anti-oxidant defense capability leads to oxidative stress [51]. A role for ROS in aging has been reinforced by various studies demonstrating that ROS levels and oxidative damage increase with chronological age, ROS production increases with inhibition of mitochondrial function, mitochondrial dysfunction increases with age and several age-related pathologies are associated with increased ROS production and oxidative stress (Figure 1) [53,55,56,57,58,59]. Although these findings suggest a tight link between ROS generation and oxidative damage during aging, whether increased ROS production is a cause or consequence of aging remained controversial. In the past decade, a number of studies have found that moderate increase in ROS levels in response to stress serves as a survival signal that extends lifespan, and that increased oxidative stress does not reduce lifespan in multiple model organisms. For example, increased ROS production was shown to extend lifespan in both yeast and *Caenorhabditis elegans* [60,61]. Reduced superoxide dismutase 2 (Sod2) activity in heterozygous *Sod2*^+/−^ mutant mice triggered enhanced oxidative DNA damage but did not accelerate aging [62]. Mice with a combined deficiency of Sod2 and glutathione peroxidase-1 (Gpx-1) antioxidant enzymes also showed increased oxidative damage but no lifespan shortening [63]. Interestingly, mtDNA mutations that do not cause oxidative stress accelerate aging in mice [42,43,64,65,66] and mtDNA is not more susceptible to oxidative damage than nuclear DNA due to its closer proximity to the site of mitochondrial ROS generation [67]. Together, these findings implicate that increased ROS generation or oxidative stress is not the primary or initial cause, but rather a consequence of aging which thus prompts a re-evaluation/reconsideration of the role of free radical theory in aging. Future studies are needed to determine whether enhanced ROS production and oxidative stress are underlying causes of mitochondrial dysfunction in various age-related pathologies or whether bioenergetic deficiency is the primary causal factor.

### 2.3. Mitochondrial Dynamics

Mitochondria are highly dynamic structures as they continuously undergo fission and fusion processes (Figure 1) that shape their morphology and regulate mitochondrial size, number and function [68]. Mitochondrial dynamics is essential for mitochondrial viability and response to changes in cellular bioenergetic status [69]. Mitochondrial fission is vital for mitotic segregation of mitochondria to daughter cells, distribution of mitochondria to subcellular locations, and mitophagy [70,71,72]. Unopposed fission leads to mitochondrial fragmentation, loss of OXPHOS function, mtDNA depletion and ROS production, which are associated with metabolic dysfunction or disease [73]. Mitochondrial fusion is essential for maintaining mitochondrial membrane potential, ATP production and maximal respiratory capacity. Unopposed fusion generates a network of hyperfused mitochondria associated with increased ATP production, reduced ROS generation and which exhibit an ability to counteract metabolic insults, protect against autophagy as well as apoptosis [73]. Changes in metabolic demand and nutrient availability regulates the rate of fission and fusion causing mitochondria to become either fragmented or hypertubular [74,75]. In mammals, mitochondrial fission is mediated by the action of dynamin-related protein (DRP1) which is recruited to mitochondria by multiple receptors (e.g., fission 1 protein (FIS1), mitochondrial fission factor (MFF), mitochondrial dynamics proteins of 49 kDa (MID49) and 51 kDa (MID51) respectively) whereas mitochondrial fusion is a two-step process that involves the action of mitofusins for outer membrane (i.e., MFN1 and MFN2) and inner membrane (i.e., optic atrophy 1 (OPA1)) fusion [68]. Ablation of mitochondrial fission and fusion proteins has been shown to cause embryonic lethality in mice [76,77,78].

In the past decade, several studies have shown that mitochondrial dynamics plays a crucial role in the regulation of mitochondrial function and metabolism, mtDNA stability, calcium homeostasis, autophagy, mitophagy as well as apoptosis [79,80,81,82,83,84,85,86,87]. Altered mitochondrial dynamics is therefore strongly linked to aging and various age-related diseases (Figure 1) [87,88]. For example, it has been documented that aging is characterized by a progressive decline in Mfn2 expression in skeletal muscle, which promotes mitochondrial dysfunction and underlies the age-related disturbances in metabolic homeostasis as well as sarcopenia [87]. Reduced expression of Mfn1 and/or Mfn2 in skeletal muscle has been linked to obesity and type 2 diabetes mellitus in both rodents and humans [81,89,90]. Furthermore, the liver-specific ablation of *Mfn2* in mice was shown to cause glucose intolerance and enhanced gluconeogenesis resulting in impaired insulin signaling and glucose homeostasis in vivo [81]. The pro-opiomelanocortin (POMC) neuronal-specific *Mfn2* ablation in mice caused hyperphagia, reduced energy expenditure, endoplasmic reticulum stress-induced leptin resistance and obesity, indicating that Mfn2 plays a vital role in maintaining systemic energy balance [91]. Interestingly, the agouti-related protein (AGRP) neuronal-specific *Mfn1* or *Mfn2* deletion in mice exhibited altered mitochondrial size and density in AGRP neurons but gained less weight in response to high-fat diet due to reduced fat mass [92]. The liver-specific *Mfn1* deletion in mice displayed a highly fragmented mitochondrial network coupled to enhanced respiratory capacity and these mice were also protected against the high-fat diet-induced insulin resistance [85]. Recently, using brown adipose tissue-specific *Mfn2* knockout mice it was demonstrated that Mfn2 is crucial for thermogenesis, and when fed a high-fat diet, these mice are protected against insulin resistance, hepatic steatosis and obesity [93,94]. Together, these findings suggest variable cell and tissue-specific effects of mitofusins in the regulation of whole-body energy metabolism that warrant further investigation. In contrast to mitofusins, increased expression of mitochondrial fission proteins in mouse skeletal muscle have been linked to obesity [90]. Thus, genetic or pharmacological inhibition of *Drp1* improved muscle insulin sensitivity and insulin signaling in obese mice, as well as conferred protection against high-fat diet-induced obesity in liver-specific *Drp1* deleted mice [95,96]. 

Alterations in mitochondrial fission and fusion machinery have also been linked to several other age-related disorders including cardiac and neurodegenerative diseases, muscle atrophy and sarcopenia. For instance, reduced Mfn1 and/or Mfn2 expression were reported to induce vascular proliferative disorders i.e., atherosclerosis and restenosis, cardiac hypertrophy, cardiomyopathy as well as cardiac failure in rodents [97,98,99,100]. Interestingly, heart-specific deletion of both *Mfn1* and *Mfn2* also conferred protection against myocardial infarction caused by acute ischemia/reperfusion injury suggesting distinct roles of mitofusins in response to acute or chronic cardiac insults [101]. Decreased Opa1 levels have been associated with cardiomyopathy and heart failure in mice [80,102], whereas enhancing Opa1 levels protected mice hearts and brain from ischemic damage [84]. Moreover, inhibition of Drp1-mediated mitochondrial fragmentation showed protection against long-term cardiac dysfunction [103] as well as neurodegeneration associated with Huntington’s disease in rodents [104]. Genetic mutations or alterations in mitochondrial fusion and fission are also linked to neuropathies [105,106], abnormal brain development, microcephaly, optic atrophy and hypoplasia [107], degeneration of cerebellum and dopaminergic neurons [108,109], as well as defective neurodevelopment, plasticity and function in various neurodegenerative disorders e.g., Alzheimer’s disease, Parkinson’s disease and Huntington’s disease [82,110,111]. Furthermore, reduced expression of mitochondrial fission and fusion proteins has been observed in muscle from rodents and humans during aging [87,112,113,114]. The skeletal muscle-specific knockdown of fusion proteins (i.e., Mfn1 and Mfn2) and overexpression of fission proteins (Drp1 and Fis1) was shown to induce muscle atrophy in mice, whereas inhibition of mitochondrial fission conferred protection against muscle atrophy in mice [79,115,116]. Together, these studies suggest that dysregulation of mitochondrial dynamics could contribute to aging and age-related pathologies. However, there are several outstanding questions that yet remain to be addressed regarding the link between mitochondrial dynamics and aging. For example, which factors cause altered expression of mitochondrial fission and fusion proteins during aging, and whether these factors are genetic or affected by environmental stimuli? Is altered mitochondrial dynamics a major cause of mitochondrial dysfunction in aged cells or tissues? Which signaling pathways regulate mitochondrial dynamics and how does their modulation affect aging-related phenotypes in model organisms? What are the physiological and metabolic consequences of perturbed mitochondrial dynamics and do different tissues respond similarly or differently to these perturbations? Whether proteins involved in mitochondrial dynamics could serve as promising candidates for promoting healthy aging and/or alleviating various age-related pathologies? Future experimental studies that are designed to address these questions would help to better understand the role of mitochondrial dynamics in aging and age-related pathologies.

### 2.4. Mitophagy and Mitochondrial Biogenesis

Autophagy is a process by which intracellular components are delivered to lysosomes for degradation and recycling [117], whereas mitophagy is a specialized form of autophagy that involves selective degradation and removal of superfluous and damaged or dysfunctional mitochondria (Figure 1) [118]. Mitophagy is highly conserved among eukaryotes and has been extensively studied in yeast, *Drosophila*, *C. elegans* and mammalian cells [20,119,120]. Mammalian cells harbor multiple mitophagy pathways that can compensate for the loss of others. One of the most characterized mitophagy pathways is phosphatase and tensin homolog (PTEN)-induced putative kinase 1 (PINK1) / Parkin-dependent pathway although mitophagy can also occur in a Parkin-independent manner [121,122,123,124,125,126]. Interestingly, the rates of mitophagy are reportedly different between and within tissues [127], and in response to different intracellular and/or environmental cues, specific mitophagy pathways are activated to maintain mitochondrial function and cell homeostasis. The canonical PINK1/Parkin-dependent mitophagy pathway is activated in response to mitochondrial damage (e.g., loss of mitochondrial membrane potential or accumulation of misfolded proteins) that involves stabilization of PINK1 on the outer mitochondrial membrane where it phosphorylates ubiquitin [128]. The ubiquitin phosphorylation recruits cytosolic E3 ubiquitin-protein ligase–Parkin to mitochondrial outer membrane which polyubiquitinates mitochondrial proteins to facilitate their association with the autophagy receptors, thereby leading to the formation of autophagosome [129,130]. The autophagosome subsequently fuses with the lysosome to promote mitochondrial degradation [120,125]. Ubiquitination of mitochondrial proteins thus identifies or tags damaged mitochondria for Parkin-mediated mitophagy, a process shown to be inhibited by deubiquitination of Parkin substrates [131]. Alternatively, PINK1 can also recruit autophagy receptors directly to mitochondria in a Parkin-independent manner to mediate mitophagy [125]. Mutations in the human *PINK1* and Parkin genes cause autosomal recessive forms of Parkinson’s disease [132,133]. Germline deletion of *Pink1*^−/−^ in mice was reported to cause mitochondrial dysfunction in the striatum at 3–4 months of age that exacerbated with chronological age and enhanced sensitivity to oxidative stress, implicating that Pink1 is critical for mitochondrial function and protection against oxidative stress [134]. Parkin^−/−^ ablation in mice was also shown to cause brain mitochondrial dysfunction, enhanced oxidative damage and metabolic abnormalities; however, these mice did not show any signs of neurodegeneration [135,136]. Notably, when Parkin^−/−^ knockout mice were crossed with the mtDNA mutator mice, the resulting mice progeny displayed an exacerbated response to mitochondrial dysfunction and degeneration of dopaminergic neurons in the substantia nigra region of the brain, suggesting that endogenous Parkin is crucial for protecting dopaminergic neurons against oxidative damage-induced cell death by enforcing mitochondrial quality control [137].

Mitochondrial fission and fusion play key roles in regulating mitophagy and quality control (Figure 1). For example, mitochondrial fission followed by selective fusion was shown to be required for the segregation of dysfunctional mitochondria and their subsequent removal by mitophagy in mammalian pancreatic β cells [138]. Disruption of mitochondrial fission protein Drp1 was found to inhibit mitophagy in mouse cardiomyocytes [139,140]. Recently, it was demonstrated that mitochondrial fission promotes selective removal of mitochondria harboring misfolded protein aggregates [141]. Accumulating evidence also suggests that PINK1 and Parkin are required for mitophagy of damaged or dysfunctional mitochondria. For example, damaged mitochondria in human dopaminergic SH-SY5Y cells or *Drosophila* were shown to promote ubiquitination of mitofusins that targeted them for degradation via the PINK1/Parkin-dependent pathway [142,143]. Expression of a mutant ubiquitin (S65A) that could not be phosphorylated by PINK1 was shown to inhibit Parkin translocation to damaged mitochondria in HeLa cells [129]. Moreover, *Mfn2* knockdown in mouse cardiac myocytes prevented Parkin translocation to depolarized mitochondria, thereby suppressing mitophagy, suggesting that Mfn2 is a mitochondrial receptor for Parkin and is critical for cardiac mitochondria quality control [100]. Despite recent research advances, the molecular mechanisms that connect mitochondrial dynamics and quality control systems remain obscure. Future studies should be directed at elucidating the mechanistic link between mitochondrial dynamics and mitophagy and determining whether it is deregulated during aging.

**Figure 2:**
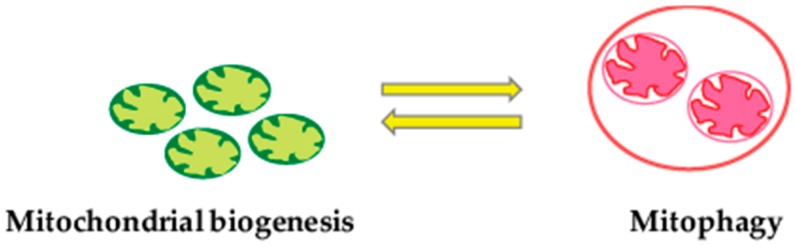
Crosstalk between mitochondrial biogenesis and mitophagy. The crosstalk or coordination between the two opposing processes i.e., selective elimination of damaged or dysfunctional mitochondria by mitophagy and generation of newly synthesized mitochondria by mitochondrial biogenesis is pivotal for the maintenance of mitochondrial energy and cellular homeostasis in response to various physiological and environmental cues.

Maintenance of mitochondrial function and cellular homeostasis not only requires selective elimination of defective mitochondria but also generation of newly synthesized mitochondria by stimulation of mitochondrial biogenesis program to maintain adequate mitochondrial mass and quality (Figure 2) [20]. Mitochondrial biogenesis is a tightly regulated process that involves coordinated transcriptional regulation of both nuclear and mitochondrial genomes to produce new mitochondria [19,144]. The transcription factors including nuclear respiratory factors i.e., NRF1 and NRF2, and transcriptional coactivators of the PPARγ coactivator-1 family i.e., PGC1α, PGC1β and PGC1 related coactivator (PRC), are key components of the complex regulatory network that orchestrates mitochondrial biogenesis [144,145]. Both NRF1 and NRF2 regulate expression of several nuclear encoded mitochondrial proteins including mtDNA transcription factors A, B1 and B2 (i.e., TFAM, TFB1M, and TFB2M), as well as proteins involved in mitochondrial respiratory chain, import machinery and heme biosynthesis [144,146]. The peroxisome proliferator-activated receptor gamma coactivator 1α (PGC1α) is a master regulator of mitochondrial biogenesis as it co-activates expression of multiple transcription factors including NRF1, NRF2 and estrogen-related receptor α (ERRα) [144,145].

The crosstalk between mitochondrial biogenesis and turnover pathways is critical for cells to adjust their pool of functional mitochondria in response to physiological or metabolic demands, stress, and other intracellular or environmental cues (Figure 2). Coordination of these two opposing processes of mitochondrial biogenesis and degradation is achieved through multiple transcriptional and post-translational regulation mechanisms [147]. For instance, increased cyclic adenosine monophosphate (cAMP) levels cause protein kinase A (PKA)-dependent activation of cAMP response element binding protein (CREB) that upregulates PGC1α expression and negatively regulates mitophagy via PKA-mediated phosphorylation and inhibition of LC3 [148,149]. In *C. elegans*, the transcription factor SKN-1 has been shown to regulate both mitophagy and mitochondrial biogenesis pathways [20]. This study demonstrated that impaired mitophagy in *C. elegans* compromises stress resistance and activates the mitochondrial retrograde signaling to induce SKN-1 expression, which in turn stimulates mitochondrial biogenesis and degradation by enhancing DCT-1 expression, suggesting a homeostatic feedback loop mechanism that integrates stress signal to coordinate induction of mitophagy and mitochondrial biogenesis pathways [20]. Additionally, besides stimulating mitochondrial biogenesis PGC1α has also been shown to enhance mitophagy and autophagy by activating the expression of transcription factor EB (TFEB)—a master regulator of lysosomal biogenesis and autophagy [150], and TFEB in turn further stimulates PGC1α expression, thereby generating a positive feedback loop mechanism to establish a balance between mitochondrial biogenesis and turnover [151]. Interestingly, a putative nutrient-sensing regulator (GCN5L1) was shown to negatively regulate both mitochondrial biogenesis and turnover pathways by inhibiting PGC1α and TFEB expression, respectively, in mouse embryonic fibroblasts [152]. Furthermore, in response to cellular stress adenosine monophosphate (AMP)-activated kinase (AMPK) has been shown to induce mitophagy via mammalian target of rapamycin (mTOR) inhibition and ULK1 activation [153,154], whereas AMPK-mediated SIRT1 phosphorylation activates PGC1α, which stimulates mitochondrial biogenesis [155]. Finally, the mitogen-activated protein kinase/extracellular-signal-regulated kinase (MAPK/ERK) pathway activation has also been shown to stimulate mitochondrial biogenesis by enhancing PGC1α expression as well as induce mitophagy in response to starvation or hypoxia-mediated cellular stress in mammalian cells [156,157]. Thus, coordination between mitophagy and mitochondrial biogenesis is pivotal in maintaining healthy mitochondrial population in eukaryotic cells in response to various environmental and intracellular cues. Deregulation of the mechanisms that coordinate mitochondrial biogenesis and turnover could lead to loss of energy and cellular homeostasis resulting in pathological conditions. 

Decline in both mitophagy and autophagy pathways are associated with aging and various age-related pathologies including neurodegenerative, cardiac and immune system disorders, hepatic dysfunction, kidney failure as well as cancer [10,158,159,160,161]. For instance, age-dependent decline in mitophagy in *C. elegans* was shown to prevent clearance of dysfunctional mitochondria and mitochondrial biogenesis resulting in accrual of mitochondrial damage accompanied with deterioration of cellular function [20,21]. Defective mitophagy has been implicated in Parkinson’s disease [16], and high levels of mtDNA deletions have been reported in the substantia nigra neurons from aged humans and patients with Parkinson’s disease [40,162]. Enhanced mitophagy accompanied by Parkin depletion with disease progression was reported in the Alzheimer’s disease patient brains, suggesting that an inadequate ability to eliminate dysfunctional mitochondria over time may lead to accrual of damaged mitochondria in neurons [163]. It has also been demonstrated that mutant huntingtin transcriptionally represses PGC1α in mice, thereby inhibiting mitochondrial biogenesis [164] and impairs mitophagy in *Drosophila* neurons by reducing the targeting of damaged mitochondria to autophagosomes [165]. The overexpression of PINK1 alleviated defective mitophagy and ameliorated neuronal integrity, ATP levels as well as adult fly survival, suggesting that PINK confers neuroprotection against mutant huntingtin in flies [165]. Interestingly, the inhibition of mitochondrial deubiquitinase USP30—an enzyme that antagonizes PINK1-Parkin-mediated mitophagy was also shown to promote mitochondrial clearance and quality control in fly neurons as well as improve mitochondrial integrity in PINK1- or Parkin-deficient flies [166]. Autophagy inhibition in mouse muscle was shown to impair neuromuscular synaptic function, muscle strength and significantly shorten the lifespan of mice [167]. Interestingly, the skeletal muscle from old mtDNA mutator mice exhibited increased mitochondrial fission and autophagy levels compared to the age-matched wild-type mice, indicating that higher autophagy in aged muscle of mtDNA mutator mice may be the cause of their sarcopenic phenotype [168]. Age-dependent decline in mitophagy/ autophagy affects cardiac physiology and alterations in mitophagy/autophagy have been implicated in various heart diseases including cardiac hypertrophy, cardiomyopathy and heart failure. For example, cardiac specific loss of autophagy-related 5 (Atg5) in mice was shown to cause cardiomyopathy and cardiac hypertrophy associated with contractile dysfunction, disorganized sarcomeres, and accrual of dysfunctional mitochondria [169]. The PINK1-Parkin mitophagy pathway was also reported to be indispensable for normal heart function as Pink1^−/−^ deficient mice displayed cardiac hypertrophy, increased oxidative stress, mitochondrial dysfunction, higher fibrosis and cardiomyocyte apoptosis [170], whereas Parkin deficient mice exhibited increased sensitivity to myocardial infarction, reduced survival, and impaired mitophagy associated with accumulation of swollen dysfunctional mitochondria [171]. Moreover, Parkin deficiency with age was shown to cause accumulation of aberrant mitochondria in mouse myocytes [172]. Furthermore, impaired mitochondrial biogenesis is also linked to myocardial hypertrophy and ischemic heart failure in humans [173]. Thus, a proper balance between mitochondrial biogenesis and clearance is essential for cardiac homeostasis. Further research is needed to identify and validate novel pharmacological targets that restore coordination or balance between mitochondrial biogenesis and turnover pathways, which might be beneficial for aging retardation and/or combating various age-related pathologies.

As discussed above, mitochondrial quality control system ensures mitochondrial integrity, function and maintenance of a healthy mitochondrial population by limiting mitochondrial damage. Failure of mitochondrial quality control measures to remove damaged or dysfunctional mitochondria impairs protein homeostasis (or proteostasis), leading to the activation of apoptotic pathways [23,174,175]. Intriguingly, mitochondrial dynamics protein machinery has been linked to the regulation of apoptosis. For example, during apoptosis Drp1 translocates from the cytosol to mitochondria and promotes mitochondrial network fragmentation, whereas dominant negative Drp1 mutant expression prevents mitochondrial fragmentation, cytochrome *c* release and apoptosis [176]. Other mitochondrial fission and fusion mediators have also been shown to regulate apoptosis including FIS1, OPA1, MFF1, MFN1 and MFN2 [177,178,179]. Interestingly, there is also a significant crosstalk between autophagy and apoptosis suggesting that these physiologically distinct processes share common targets and/or pathways [180]. The mitochondrial proteostasis system acts to ensure that proteins are maintained in their native folded state, while unfolded, misfolded or unassembled proteins are targeted for degradation by various proteases (e.g., AAA, Clp and Lon proteases) [23,181]. Aging and certain age-related diseases have been linked to proteostasis deficiency [2,182,183]. For instance, the activity of mitochondrial matrix Lon protease (LonP) which largely degrades oxidized proteins was found to be reduced with age in mice [184]. Furthermore, loss of proteostasis could result in accrual of unfolded, misfolded or aggregated proteins, leading to the development of age-related neurodegenerative disorders (e.g., Alzheimer’s disease, Parkinson’s disease, Huntington’s disease) and cataracts [182]. The mitochondrial proteostasis and programmed cell death activation pathways during aging will not be discussed in depth here (see recent reviews that have covered these topics in detail [2,181,182,183,185,186,187,188,189,190]).

### 2.5. Mitochondria-Mediated Longevity Pathways

Mitochondria communicate changes in their functional and metabolic state to the nucleus through activation of a retrograde signaling pathway in response to various intracellular and environmental cues as well as during aging. Mitochondrial retrograde signaling pathway is thus an adaptive response that elicits coordinate expression of a broad array of nuclear genes in an attempt to restore mitochondrial energy and cellular homeostasis that promotes cell survival [191]. Studies from multiple model organisms indicated that loss in mitochondrial membrane potential, increased ROS generation and mitochondrial metabolites (e.g., ATP, nicotinamide adenine dinucleotide (NAD^+^), acetyl coenzyme A (acetyl-CoA)) could serve as signal transducers to activate the mitochondrial–nuclear retrograde response [192,193]. Notably, the retrograde signaling pathway in yeast was initially shown to extend the cellular replicative lifespan [194]. Subsequently, many studies reported that mild respiratory chain impairment leading to low levels of ROS increase induces an adaptive or hormetic response which enhances cellular stress resistance and systemic defense—concept of ‘mitohormesis’ [195,196,197]. Mitohormesis has been shown to extend lifespan and promote health in multiple model organisms [195,196,197]. Mitochondrial stress that occurs due to accumulation of unfolded, misfolded or unassembled proteins elicits retrograde signaling that induces a distinct nuclear transcriptional response resulting in the induction of mitochondria-specific protein chaperones such as heat shock protein (HSP) factors. This retrograde signaling pathway is the mitochondrial unfolded protein response (UPR^mt^) pathway, which is an adaptive response to the mitochondrial proteotoxic stress. The UPR^mt^ pathway was elegantly described in *C. elegans* and has also been shown to extend lifespan in diverse model organisms including yeast, worms, flies and mice [191,198]. However, the precise mechanisms by which UPR^mt^ pathway promotes longevity remain poorly understood. Moreover, the molecular components including sensors and signal transduction effectors as well as the potential mechanisms that regulate UPR^mt^ pathway in mammals are yet to be completely uncovered. Interestingly, the mitochondrial stress response can also be elicited in distal tissues/organs that are not affected by the initial stress event through soluble factors or ‘mitokines’ in a non-cell-autonomous manner as demonstrated in *C. elegans* [199]. Multiple studies have recently identified new mitokines that are produced and secreted in response to mitochondrial stress i.e., the fibroblast growth factor 21 (FGF 21) and growth differentiation factor 15 (GDF15) [200,201]. These mitokines act on different tissues and organs to exert their systemic beneficial effects such as improved glucose tolerance, resistance to diet-induced obesity and weight loss [200]. The retrograde signaling pathway interacts with various other signaling pathways including the energy or nutrient sensing pathways such as AMPK, mTOR and insulin/insulin-like growth factor signaling (IIS) and is also linked to mitophagy and autophagy. 

Emerging evidence suggests that enhanced mitophagy and autophagy pathways promote healthy aging as well as delay the onset and progression of various age-related pathologies [202]. For instance, enhanced mitophagy mediated by ubiquitous or neuron-specific overexpression of Parkin in *Drosophila melanogaster* significantly reduced age-associated proteotoxicity and extended the organismal lifespan [203]. Moreover, a natural compound, urolithin A found in pomegranates was recently shown to extend lifespan in *C. elegans* as well as enhance muscle function in rodents by inducing mitophagy [204]. Other natural compounds that exert beneficial effects on cellular energy metabolism and organismal health and lifespan include NAD^+^, resveratrol and spermidine [3,8,205,206,207]. Besides natural compounds, interventions such as dietary restriction, physical exercise and certain pharmacological drugs are known to retard aging and attenuate age-associated functional decline, thus promoting health and extending lifespan in diverse model organisms [3,208,209,210]. Beneficial effects of caloric restriction (CR) in aging retardation have been observed in yeast, flies, worms, mice as well as non-human primates, and recent studies suggest that autophagy is required for lifespan extension by CR in multiple model organisms [3,211,212,213]. Interestingly, the health benefits of physical exercise partially overlap with CR in terms of improving cellular metabolism, increasing NAD^+^ availability that activates sirtuins, stimulating PGC1α expression, and facilitating mitochondrial quality control [214,215,216]. Finally, pharmacological drugs such as rapamycin (mTOR inhibitor) and metformin (AMPK activator) also extend lifespan in model organisms and confer protection against several age-related pathologies [217,218]. Notably, the longevity mediating mitochondrial signaling pathways are highly interconnected and/or cross-regulated, resulting in a complex network that modulates common targets, which could be difficult to separate [219,220]. For instance, CR has been shown to ameliorate oxidative stress and extend organismal lifespan by modulating AMPK, mTOR, IIS and sirtuins signaling pathways [221]. Autophagy, which is required for lifespan extension effects mediated by CR, is also regulated by AMPK, mTOR, SIRT1 and FOXO signaling pathways [222,223,224]. Modulation of AMPK and mTOR signaling pathways could thus combine the simultaneous induction of mitochondrial biogenesis and autophagy/mitophagy to retard aging, promote longevity and protect against various age-related pathologies. Future studies are required to uncover the molecular connections and mechanisms that underlie the cross-regulation and interdependence of multiple signaling pathways during aging.

## 3. Conclusions and Perspective

Mitochondria are central to the regulation of energy metabolism and cellular homeostasis due to their principal role in bioenergetics, ROS production, ion homeostasis, apoptosis and signal transduction. These organelles are highly dynamic and can reprogram metabolism in response to various environmental and intracellular cues. Mitochondrial functional decline and accrual of damaged mitochondria in various tissues is associated with aging. However, the functional relevance of somatic mtDNA mutations in the aging process requires further clarification as they do not appear to be the main cause of aging. The relationship between ROS and aging is far more complex than it was originally perceived, and increased ROS or oxidative stress is also not the primary trigger of aging. In fact, moderate increase in ROS levels in response to caloric restriction, physical exercise or mild mitochondrial dysfunction promotes health and longevity in multiple model organisms—concept of mitohormesis [225]. Additional studies are however required to test whether similar to animal models mitohormesis also works in humans, and whether there is a precise threshold for mitochondrial impairment that could distinguish a hormetic response from pathological state. Maintenance of proteostasis is essential for healthy aging and could aid in the prevention of age-related disorders caused by protein misfolding. In fact, pharmacological drugs targeting multiple components of the proteostasis machinery are being tested in clinical trials [226].

Perturbations in mitochondrial function, biogenesis and dynamics impair cellular homeostasis and trigger mitochondrial quality control mechanisms. Altered mitochondrial dynamics and quality control promote accumulation of damaged mitochondria that contributes to aging and several age-related pathologies. Thus, strategies that effectively improve or rescue the defect in mitochondrial dynamics and quality control may be beneficial in combating aging and age-associated diseases. Towards this goal, development of small molecules that can therapeutically augment mitochondrial biogenesis, and pharmacological induction of mitophagy through use of mitophagy-activating compounds or NAD^+^ supplementation might be beneficial for patients with age-related disorders and to combat aging. Additionally, agents that modulate the activity of PINK1, Parkin and USP30 could also promote mitophagy and prove beneficial in alleviating age-associated pathologies. In the upcoming years, further studies are needed to develop and test the potential effects of mitophagy modulators on cellular energy metabolism as well as organismal health and lifespan. Notably, mitophagy stimulation could be both beneficial or detrimental for tissue and organismal homeostasis based on the specific cellular bioenergetics requirement under healthy and diseased states. Therefore, effective new therapeutic strategies should involve coordinate induction of both mitophagy and mitochondrial biogenesis in order to maintain healthy mitochondrial population in cells. Finally, interventional studies will be necessary to test how mitophagy and mitochondrial biogenesis-inducing compounds impact human physiology and test their potential therapeutic efficacy for future clinical applications.
